# Flipping the dogma – phosphatidylserine in non-apoptotic cell death

**DOI:** 10.1186/s12964-019-0437-0

**Published:** 2019-10-29

**Authors:** Inbar Shlomovitz, Mary Speir, Motti Gerlic

**Affiliations:** 1 Department of Clinical Microbiology and Immunology, Sackler Faculty of Medicine, Tel Aviv University, Tel Aviv, Israel; 2 Centre for Innate Immunity and Infectious Diseases, Hudson Institute of Medical Research, Clayton, VIC 3168 Australia; 3 Department of Molecular and Translational Science, Monash University, Clayton, VIC 3800 Australia

**Keywords:** Cell death, Inflammation, Apoptosis, Necroptosis, Phosphatidylserine, AnnexinV, Phagocytosis, Extracellular vesicles, ESCRT, Efferocytosis

## Abstract

**Abstract:**

The exposure of phosphatidylserine (PS) on the outer plasma membrane has long been considered a unique feature of apoptotic cells. Together with other “eat me” signals, it enables the recognition and phagocytosis of dying cells (efferocytosis), helping to explain the immunologically-silent nature of apoptosis. Recently, however, PS exposure has also been reported in non-apoptotic forms of regulated inflammatory cell death, such as necroptosis, challenging previous dogma. In this review, we outline the evidence for PS exposure in non-apoptotic cells and extracellular vesicles (EVs), and discuss possible mechanisms based on our knowledge of apoptotic-PS exposure. In addition, we examine the outcomes of non-apoptotic PS exposure, including the reversibility of cell death, efferocytosis, and consequent inflammation. By examining PS biology, we challenge the established approach of distinguishing apoptosis from other cell death pathways by AnnexinV staining of PS externalization. Finally, we re-evaluate how PS exposure is thought to define apoptosis as an immunologically silent process distinct from other non-apoptotic and inflammatory cell death pathways. Ultimately, we suggest that a complete understanding of how regulated cell death processes affect the immune system is far from being fully elucidated.

**Graphical abstract:**

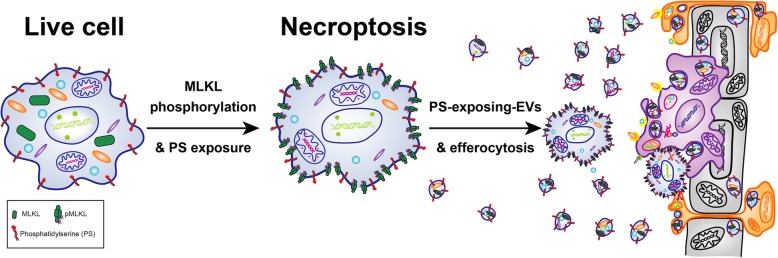

## Plain English summary

For a long time, it has been considered that when cells are programed to die via a mechanism known as apoptosis, they alarm neighboring cells using “eat me” signals to facilitate their clearance from our body. Recently, it has been reported that even when cells die via a regulated but non-apoptotic pathway (termed necroptosis) they still possess similar “eat me” signals to apoptotic cells. In this review, we outline the evidence for these “eat me” signals in non-apoptotic cell death, and discuss the possible mechanisms and implications of such signals.

### Background

Cell death is central to physiological homeostasis; the balance between cellular differentiation, proliferation, and death underpins all aspects of biology, including embryogenesis, organ function, immune responsivity, and tumorigenesis [1]. Originally, cell death was divided into two basic forms, termed apoptosis (programmed cell death) and necrosis (accidental cell death), which were distinguished primarily by their morphology as observed by pathologists. In the last two decades, however, the cell death field has expanded to include upwards of 10 distinct, although sometimes overlapping, pathways [2].

### Apoptosis

Defined in 1972, apoptosis was the first form of regulated cell death (RCD) to be discovered [3]. Apoptosis is executed either by intrinsic or extrinsic pathways, which ultimately lead to the activation of a family of cysteine-dependent aspartate-specific proteases called caspases [4–6]. In the extrinsic pathway, ligation of death ligands (e.g., TNF-related apoptosis-inducing ligand (TRAIL) [7], tumor necrosis factor (TNF) [8], or Fas ligand (FASL) [9]) to their respective death receptors recruits and activates the initiator caspases-8 and -10 in an interaction mediated by death domain–containing adaptor proteins, e.g., Fas-associated protein with death domain, FADD [10]. In the intrinsic, or mitochondrial, pathway, cellular stress modifies the balance between pro- and anti-apoptotic B-cell lymphoma-2 (Bcl-2) family members, releasing pro-apoptotic BAX and BAK to induce mitochondrial outer membrane permeabilization (MOMP). Cytochrome-c release following mitochondrial damage activates the initiator caspase-9 [11, 12], which then cleaves the effector caspases-3, − 6, and − 7 to execute apoptosis [13, 14]. Hallmarks of apoptotic cell death are cell shrinkage, chromatin condensation (pyknosis) [15], DNA fragmentation [16], plasma membrane blebbing [17], and the shedding of apoptotic bodies [18–20]. Another main feature is the exposure of phosphatidylserine (PS) on the outer plasma membrane, which, among other “eat me” signals, results in the phagocytosis and clearance of apoptotic cells and bodies without the release of pro-inflammatory molecules [21]. Hence, apoptosis has always been classified as an immunologically silent form of cell death [22].

### Necrosis

The term necrosis was originally used by Rudolf Virchow to describe tissue breakdown while configuration was conserved [23]. Necrosis is now considered to be a trauma-induced form of accidental cell death (ACD) [2]. Morphologically, necrosis is characterized by the swelling of the cell (oncosis) and its organelles, as well as by permeabilization of the plasma membrane that releases cellular contents into the extracellular space to trigger inflammation [20]. While originally considered to be unprogrammed, necrosis is now understood to also be a regulated process that can be genetically and chemically manipulated. Many pathways of regulated necrosis have now been discovered, including necroptosis, pyroptosis, mitochondrial permeability transition (MPT)-driven necrosis, ferroptosis, parthanatos, and NETosis [2]. While these pathways represent a huge and ongoing field of investigation, this review will focus primarily on necroptosis within the context of PS biology.

### Necroptosis

**Fig. 1:**
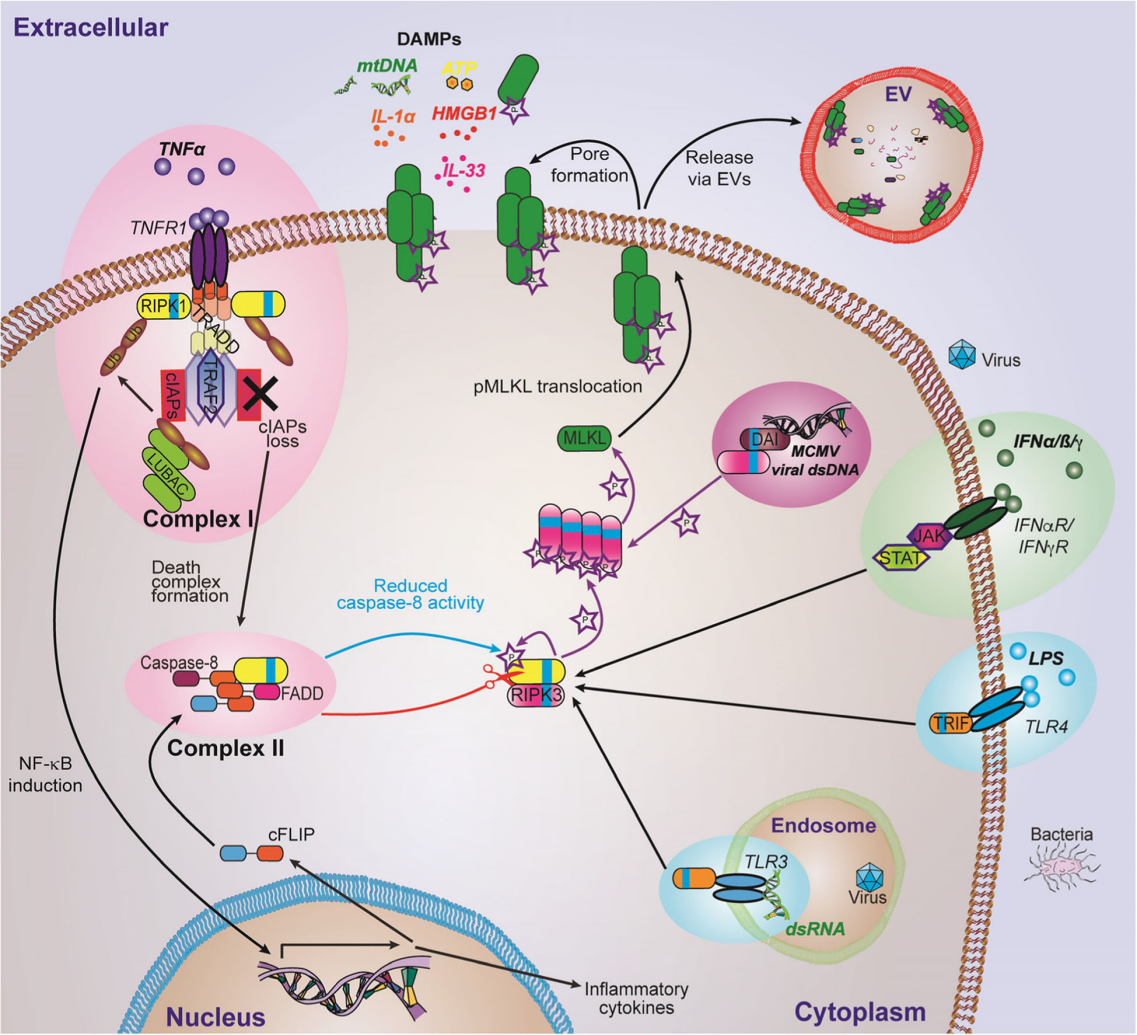
Necroptosis molecular pathway. Necroptotic cell death can be triggered by numerous factors, including death receptors, TLRs, and intracellular receptors. The ligation of TNF to its receptor (TNFR1) recruits TNFR type 1-associated via death domain (TRADD) and RIPK1 via their death domain (DD) (pink ellipse). TRADD recruits TNF receptor associated factor 2 (TRAF2) and cellular inhibitors of apoptosis (cIAPs) to collectively form complex I, together with the linear ubiquitin chain assembly complex (LUBAC). In complex I, RIPK1 is ubiquitylated to induce nuclear factor kappa-light-chain enhancer of activated B cells (NF-kB) nuclear translocation and signaling. This signaling results in the expression of inflammatory cytokines and pro-survival proteins, such as c-FLIP. When complex I activity is impaired, or following TNFR1 endocytosis, the assembly of a RIPK1/caspase-8/FADD/c-FLIP cytosolic complex, complex II, can occur. Caspase-8, in complex with c-FLIP, cleaves and inactivates RIPK1 and RIPK3. When caspase-8 activity is blocked, phosphorylation and oligomerization of RIPK3 leads to necroptosis by inducing phosphorylation of MLKL followed by its translocation to the cell membrane. The cellular contents released from necroptotic cells can serve as DAMPs to further induce inflammation. Similarly, when caspase-8 activity is blocked, necroptosis can also be induced by interferons (IFNs) (green ellipse), TLRs (blue ellipse), and DNA-dependent activator of IFN-regulatory factors (DAI) (purple ellipse). IFNs stimulate Janus kinase (JAK)-signal transducer and activator of transcription (STAT) signaling upon ligation of IFN receptors (IFNRs) resulting in RIPK1 and/or RIPK3 activation. TLRs can recruit RIPK3 via TIR-domain-containing adaptor-inducing interferon- *β* (TRIF) upon ligation by lipopolysaccharides (LPS) (for TLR4) or dsRNA (for TLR3). DAI directly interacts with RIPK3 via a RHIM-RHIM interaction upon sensing of dsDNA

Necroptosis is the most characterized form of regulated necrosis. Necroptosis was originally defined in the year 2000 as a receptor-interacting serine/threonine-protein kinase 1 (RIPK1)-dependent, caspase-independent form of cell death [24]. However, since a RIPK1-independent necroptotic pathway was latter discovered [25–27], necroptosis is now defined as a receptor-interacting serine/threonine-protein kinase 3 (RIPK3)−/mixed lineage kinase domain-like (MLKL)-dependent, caspase-independent form of cell death [28, 29]. While various factors, such as death receptors, Toll-like receptors (TLRs), and intracellular receptors, can activate necroptosis, they all share one common feature, which is the need for prior inhibition of caspase-8. Otherwise, caspase-8, in complex with cellular FLICE (FADD-like IL-1β-converting enzyme)-inhibitory protein (c-FLIP), cleaves and inactivates RIPK1 and RIPK3 [30–36]. Once caspase-8 activity is blocked, however, extra- and intracellular signals trigger auto- and trans-phosphorylation between RIPK1 and RIPK3, leading to the aggregation and phosphorylation of MLKL by RIPK3 [31, 37–39]. This culminates in the translocation of phosphorylated MLKL (pMLKL) to the plasma membrane where it compromises membrane integrity, resulting in necroptosis [40–42] (Fig. 1). As with necrosis, necroptosis is characterized by cell swelling and membrane permeabilization resulting in the release of danger associated molecular patterns (DAMPs) and consequent inflammation [25, 28, 43, 44]. Necroptosis can be prevented genetically by the depletion of RIPK3 or MLKL, as well as chemically by the inhibition of RIPK1 kinase activity [45, 46], RIPK3 kinase activity [47], or MLKL necroptotic activity [40, 48].

Similar to apoptosis, necroptosis is also important in host immune defense against various pathogens. Thus, it is not surprising that some viruses have developed factors that inhibit necroptosis as part of their virulence strategy [49]. Among these are vaccinia virus [50], cytomegalovirus (CMV) [51, 52], Epstein-Barr virus (EBV) [53], and Influenza A virus [54, 55]. Herpes simplex virus (HSV)-1 and − 2 inhibit necroptosis in human cells [56], while inducing necroptosis in murine cells, which are not their natural host [57, 58]. Bacteria, such as *Salmonella enterica* [59], *Mycobacterium tuberculosis* [60], and *Staphylococcus aureus* [61–63] induce necroptosis, while the enteropathogenic *Escherichia coli* (EPEC)-effector, EspL, directly degrades components of necroptotic signaling [64]. Both the complex role and the relevance of necroptosis in host-pathogen interactions are currently an area of intensive study [43, 65–67].

Necroptosis has also been suggested to play a role in various inflammatory pathologies, such as atherosclerosis [68], ischemia-reperfusion renal injury [69], cerulein-induce acute pancreatitis [31], neurodegenerative diseases, such as amyotrophic lateral sclerosis (ALS) [70], multiple sclerosis (MS) [71], and Alzheimer’s disease (AD) [72, 73], as well as many others. In most cases, it is still unclear whether the non-necroptotic roles of RIPK1 and RIPK3, rather than their execution of cell death, underlie disease pathology [74, 75].

### Cell death and inflammation

While the Roman Cornelius Celsus defined the four cardinal signs of inflammation (heat, redness, swelling, and pain) in the first century AD, it was not until the nineteenth century that advances in histopathology enabled Rudolf Virchow to describe the association between inflammation and tissue damage seen in necrosis. Developing technologies have now shed light on the underlying mechanism, involving cytokine and chemokine secretion, immune cell recruitment, and increased blood vessel permeability [76–78]. Inflammation is now understood to facilitate pathogen elimination and wound healing [79]. However, when not properly controlled, an excessive immune response may result in inflammatory pathology and tissue damage [80].

The inflammation-provoking agent may be either foreign or endogenous. Foreign agents are usually non-self molecules associated with a pathogen and are referred to as pathogen associated molecular patterns (PAMPs). In contrast, endogenous agents are intracellular molecules released by damaged cells and are thus referred to as danger associated molecular patterns (DAMPs). Polly Matzinger challenged the long-lived self/non-self model of immunity by proposing that the immune system is context specific, recognizing and responding to danger, rather than pathogens alone [28, 80]. Cell death and the release of cellular contents are now known to be major drivers of inflammation [81–83].

### Non-apoptotic PS exposure

The plasma membrane of viable cells exhibits phospholipid asymmetry, as phosphatidylcholine and sphingomyelin are predominantly on the outer leaflet and most phosphatidylethanolamine (PE) and phosphatidylserine (PS) are in the inner leaflet [84]. The exposure of PS on the outer leaflet of early apoptotic cells was reported back in 1992 [21]. As it was already known that the anticoagulant AnnexinV binds to negatively charged phospholipids like PS [85], it became a tool for the detection of PS-exposing apoptosing cells [86–91]. Today, it is still used as a marker for early apoptosis and is commercially distributed as a definitive tool to distinguish apoptotic from necrotic cells, mainly by flow cytometry [92–96].

Relying on this method to define apoptotic cells is problematic, however, as many groups have now also reported PS exposure in non-apoptotic cells. Krysko et al. have used immunogold labeling to detect PS on the outer plasma membrane during oncosis, the early stage of primary necrosis in which cells swell [97], while Ferraro-Peyret et al. have reported that apoptotic peripheral blood lymphocytes can expose PS in a caspase-independent manner [98]. In support, Sawai and Domae have shown that the pan-caspase inhibitor, z-VAD-fmk (zVAD), does not prevent AnnexinV staining and cell death in U937 cells treated with the apoptotic stimuli, TNF-α and the protein translation inhibitor cycloheximide. Together, these reports indicate that necrotic cells cannot be distinguished from apoptotic cells using AnnexinV staining alone [99].

With advancements in our understanding of caspase-independent RCD, many of these models might now be recognized as regulated necroptosis, rather than simple necrosis. For example, Krysko et al. induced death by treating a caspase-8-deficient, bcl-2 overexpressing cell line with dsRNA. Ferraro-Peyret et al. also used zVAD prior to adding an intrinsic apoptotic stimulus, either etoposide, staurosporine, or IL-2 withdrawal. Sawai and Domae added the RIPK1 inhibitor necrostatin-1 to block PS exposure and cell death in the zVAD-, TNF-α-, and cycloheximide-treated U937 cells, strongly implying RIPK1 involvement. Consistent with this, Brouckaert et al. showed that TNF-α treated-, i.e.*,* necrotic, L929 cells are also phagocytosed in a PS-dependent manner [100], while in the nematode *Caenorhabditis elegans*, necrotic touch neurons have also been shown to expose PS [101].

Recently, we and others have demonstrated and characterized PS exposure in well-established models of necroptosis that are currently in use. Gong et al. used either RIPK3 or MLKL fused into the binding domain of FKBP-12 (Fv). These dimerizable proteins rapidly aggregate upon addition of a dimerizer, resulting in a coordinate activation and necroptosis without the need for caspase inhibition. Using this system in NIH 3T3 cells and mouse embryonic fibroblasts (MEFs), they have shown that necroptotic PS externalization occurs prior to the loss of plasma membrane integrity [102]. In our lab, we induce necroptosis in L929, HaCaT, and U937 cells using a combination of TNF-α, a second mitochondria-derived activator of caspases (SMAC) mimetic and zVAD (denoted here as TSZ) and observe the same phenomenon [103]. PS exposure has also been observed shortly before plasma membrane rupture during pyroptosis, an inflammasome−/gasdermin-D-dependent RCD that results in the cleavage and release of IL-1*β* and IL-18 [104]. In agreement, Jurkat cells were recently shown to expose PS and be phagocytosed following death by either Fas-induced apoptosis, TNF-α-induced necroptosis, or RSL3 (a glutathione peroxidase 4, GPX4, inhibitor)-induced ferroptosis [105]. In addition, it was very recently reported that necroptosis induction by IFN-*γ* in caspase-8 deficient MEFs also resulted in a long-term PS exposure before cell death execution [106]. Overall, these findings challenge the canonical approach of distinguishing apoptosis from other cell death pathways by AnnexinV staining of PS externalization before membrane rupture [107].

### Machinery of apoptotic vs non-apoptotic PS exposure

While the externalization of PS during apoptosis has long been known, the underlying molecular mechanism was elucidated only in the last decade. In a healthy cell, plasma membrane asymmetry is maintained by ATP-dependent aminophospholipid translocases or flippases that transport PS and PE to the inner leaflet of the lipid bilayer against a concentration gradient. Among various candidates, the type IV P-type ATPase (P4-ATPase) family members ATP11C and ATP11A, and their chaperone CDC50A, were found to be important for this flip [108]. While ATP11A and ATP11C deficiency decreased flippase activity without abolishing the asymmetry, CDC50A-deficient cells continually expose PS, suggesting that other molecules might also contribute. Given the established asymmetry, flippase inactivation is inadequate for rapid PS exposure, as passive translocation is too slow. Specific molecules, including transmembrane protein 16F (TMEM16F) and XK-related protein 8 (XKR8), have been found to non-specifically transport phospholipids between the lipid bilayer, and are therefore defined as phospholipid scramblases [109, 110].

PS exposure is blocked in the presence of a caspase inhibitor in anti-FAS-treated Jurkat cells, indicating PS externalization during apoptosis is caspase-dependent in these cells [111]. Indeed, the phospholipid scramblase, XKR8, is cleaved by caspase-3 during apoptosis, resulting in its dimerization and irreversible activation [112]. Cells that express caspase-resistant XKR8, or totally lack it, do not expose PS during apoptosis. Interestingly, the flippases, ATP11A and ATP11C, also contain caspase recognition sites. Cells with caspase-resistant ATP11A/C do not expose PS during apoptosis, indicating a requirement for their irreversible inactivation by caspases [108].

In contrast, TMEM16F scramblase activity is calcium-dependent, and is dispensable for lipid scrambling during apoptosis [113]. Activated platelets and lymphocytes expose PS in a Ca^2+^-dependent manner, for which TMEM16F is also essential. High Ca^2+^ levels inhibit P4-ATPase, hence flippase inhibition might also contribute in this setting [114]. Taken together, these findings distinguish the caspase-dependent mechanism of apoptotic PS exposure in which ATP11A/C are inactivated and XKR8 is activated, from PS-exposure mediated by Ca^2+^ influx.

The key players in PS exposure during necroptosis have not yet been elucidated. Using the dimerizable RIPK3 and MLKL systems described above, Gong et al. have shown that MLKL activation leads to PS exposure independently of RIPK3 and caspase activity [102]. In support of this, blocking the translocation of human pMLKL to the plasma membrane using necrosulfonamide (NSA) prevents necroptotic-PS exposure and cell death [103]. Necroptosis induces a minor and transient oscillatory rise in intracellular Ca^2+^ that is accompanied by a rectifying Cl^−^ efflux downstream of TMEM16F activation. However, neither TMEM16F knockdown, nor inhibition, affect necroptotic cell death [115]. The elevation in intracellular Ca^2+^ levels was shown to be a consequence, rather than a requirement, of MLKL activation. Although PS exposure follows the MLKL-dependent Ca^2+^ influx, it is not prevented in the absence of extracellular Ca^2+^ [116]. In addition, TMEM16F is not necessary for this PS exposure [102]. However, extracellular Ca^2+^ depletion inhibits plasma membrane breakdown, suggesting that these cells are primed to die but are “trapped” without a concomitant increase in intracellular Ca^2+^. Interestingly, intracellular Ca^2+^ levels also eventually increase when cells are cultured in Ca^2+^-free medium, suggesting that intracellular pools of Ca^2+^, in the endoplasmic reticulum (ER) for example, might ultimately supply the Ca^2+^ ions. In support, although in some cell lines it appears that cell death is totally blocked in the absence of extracellular Ca^2+^ within the time-frame examined, in others it is only delayed [116].

**Fig. 2:**
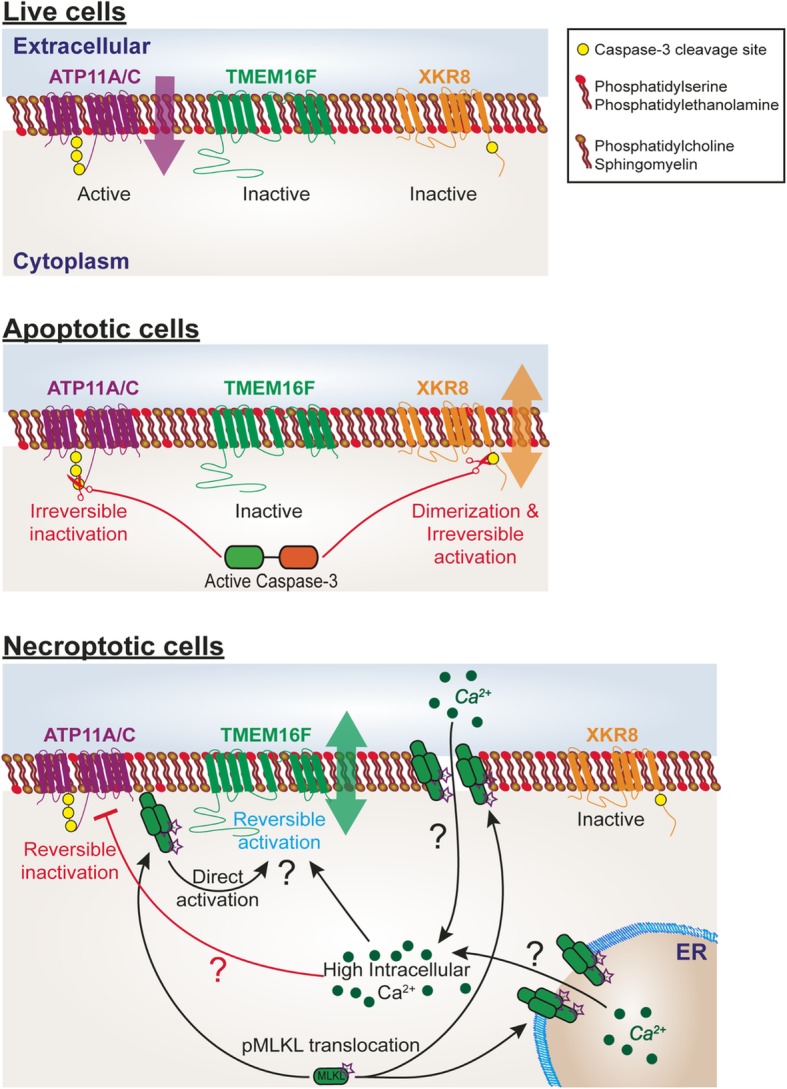
Mechanism of phosphatidylserine (PS) exposure during apoptosis and necroptosis. In live cells, the flippases, ATP11A and ATP11C, transport PS and phosphatidylethanolamine (PE) to the inner leaflet of the lipid bilayer against a concentration gradient. In apoptotic cells, active caspase-3 cleaves the phospholipid scramblase, XKR8, resulting in its dimerization and irreversible activation. In addition, caspase-3 cleaves ATP11A/C into an irreversible inactive state. The mechanism of PS exposure during necroptosis has not been elucidated. We hypothesized that pMLKL translocation-mediated increase in intracellular Ca^2+^, from either the extracellular space or the endoplasmic reticulum (ER), activates the calcium-dependent scramblase, TMEM16F, and irreversibly inactivates the flippases, ATP11A/C. pMLKL, when directly associated with the plasma membrane, might also possess the ability to directly effect TMEM16F activity, as well as other yet unknown scramblases

In agreement, Ousingsawat et al. have demonstrated that, during necroptosis, intracellular Ca^2+^ influx originates from the ER, and is thus independent of extracellular Ca^2+^ levels [115]. These data suggest that TMEM16F is being activated by the increase in intracellular Ca^2+^ during necroptosis and, hence, may have some redundant role in necroptotic PS exposure together with one, or more, as-yet unknown scramblases. However, this mechanism is not essential for subsequent cell death. Nevertheless, simultaneous staining with the Ca^2+^ sensor, GCaMP3, and MFG-E8, which does not require Ca^2+^ for PS staining, might confirm whether intracellular Ca^2+^ is needed, or not, for necroptotic PS-exposure. In addition, since PS exposure immediately follows MLKL activation and pMLKL is directly associated with the plasma membrane, MLKL might possess the ability to directly effect scramblase [102, 117] (Fig. 2). In support, *Mlkl*^*D139V/D139V*^ neonates, which carry a missense mutation results in spontaneously activated MLKL, were recently reported to demonstrate increased AnnexinV binding in some hematopoietic progenitor populations [118].

Of note, when cell death is induced by overexpression of gasdermin-D (the terminal, pore-forming executer of pyroptosis), knockdown of TMEM16F inhibits Ca^2+^-mediated PS exposure and cell death [119]. Similarly, in *Caenorhabditis elegans*, the nematode homolog of TMEM16F, anoctamin homolog-1 (ANOH-1), was found to be essential for PS exposure and phagocytosis of necrotic, but not apoptotic, cells. These results suggest a role for TMEM16F in non-apoptotic PS exposure. To add to the complexity, ANOH-1 acts in parallel to CED-7, a member of the ATP-binding cassette (ABC) transporter family, which is also required for PS exposure in apoptosis [101]. Taken together, these observations highlight that the role of Ca^2+^, caspases, flippases, and scramblases in PS exposure is specific to the type of cell death, and that new discoveries regarding the machinery and mechanism of non-apoptotic PS exposure are yet to come.

### Not just the cells - PS positive necroptotic extracellular vesicles

Focusing in on PS exposure during necroptosis, we and others have realized that this phenomenon is not restricted to necroptotic cells alone. As with apoptotic cells that form PS-exposing apoptotic bodies to facilitate their recognition and phagocytosis [95], necroptotic cells also release PS-exposing extracellular vesicles (EVs), here referred to as “necroptotic bodies”. Necroptotic bodies are smaller in size than their apoptotic counterparts (0.1–0.8 *μm* versus 0.5–2 *μm*, respectively), contain pMLKL, endosomal sorting complexes required for transport (ESCRT) family members and other proteins, and have less DNA content than the apoptotic bodies [103, 120, 121].

Using dimerizable RIPK3 and MLKL, the formation of AnnexinV+ necroptotic bodies has been reported to be rapid and dependent on MLKL activation. The fact that these bodies did not contain proteins, in this experimental system, might arise from the rapid and exogenous activation of necroptosis using the dimerizer, which bypasses the full molecular signaling pathway [102]. The ESCRT machinery comprises a group of proteins that assembles to facilitate the transportation of proteins in endosomes, multivesicular body formation, and budding [122]. The ESCRTIII components, CHMP2A and CHMP4B, translocate from the cytosol and colocalize with active MLKL near the plasma membrane during necroptosis, suggesting that they may have a role in the shedding of PS-exposing necroptotic bodies. In support, silencing of CHMP2A and CHMP4B reduced the formation and release of necroptotic bodies in both human and murine cells [102, 116, 121].

### Commitment issues – are PS-exposing necroptotic cells committed to die?

As discussed above, PS exposure during apoptosis is caspase-dependent. With more than 500 substrates, activated effector caspases are responsible for nuclear and Golgi fragmentation, chromatin condensation, DNA cleavage and degradation, and plasma membrane blebbing, all of which together promote irreversible cell death [123, 124]. Despite this, immortalized cells can be rescued from very late apoptosis, even though they expose PS [125]. This phenomenon is called anastasis, or apoptotic recovery [126]. Similarly, and maybe even more privileged by their caspase-independency, PS-exposing necroptotic cells are also not obliged to die. For example, the addition of NSA to isolated PS-exposing necroptotic cells (sorted AnnexinV-single positive U937, Jurkat, or HT-29 cells) resulted in an increase in the live cell population (AnnexinV-) over 24 h [102, 103].

Facilitating study of this phenomenon, necroptosis induced in the dimerizable RIPK3- or MLKL-expressing cells can be rapidly deactivated by the addition of a competitive inhibitor, termed a “washout ligand”. Isolated PS-exposing necroptotic cells in which RIPK3 or MLKL were inactivated by this method exhibit dephosphorylated MLKL, re-established PS asymmetry, basal intracellular Ca^2+^ levels, normal morphology, culture surface reattachment, and robust growth. These recovered cells are as susceptible to a new necroptotic stimulus as their parent cells, but appear to have a unique pattern of gene regulation, with enrichment in the fibroblast growth factor receptor (FGFR) and Gap junction pathways [116, 126].

The necroptosis survivors also show higher expression of several ESCRT components. The ESCRTIII machinery functions by shedding wounded membrane components as ‘bubbles’ in an intracellular Ca^2+^-dependent manner to maintain plasma membrane integrity [127–129], and is important for plasma membrane repair in response to diverse stimuli. Loss of ESCRT machinery components appears to compromise the recovery of PS-exposing necroptotic cells. For example, silencing of CHMP2A decreased the ability of resuscitated cells to form tumors when injected into mice. In addition, a specific clone of dimerizable RIPK3-expressing immortalized macrophages that was resistant to RIPK3 activation showed pMLKL and extensive formation of AnnexinV+ bubbles upon dimerizer treatment. Silencing of the ESCRTIII member, CHMP2A, drastically increased the susceptibility of these cells to necroptosis [102]. Overall, these data strongly indicate that the ESCRTIII machinery is essential for necroptosis recovery.

In support, bone marrow-derived dendritic cells (BMDCs) demonstrate slower and reduced cell death in response to RIPK3 activation in comparison to bone marrow-derived macrophages (BMDMs) and HT-29 cells. In alignment with the concept of shedding damaged membrane components to delay or prevent necroptosis, pMLKL under these conditions was detectable only in the secreted EVs, but not inside the BMDCs themselves. In addition, silencing of two proteins required for EVs release (Rab27a and Rab27b) increased the sensitivity of BMDCs to RIPK3-mediated cell death [121]. Hence, MLKL-mediated Ca^2+^ influx might promote PS exposure and recruit ESCRTIII, leading to the shedding of damaged PS-exposing membrane as bubbles and allowing the cell to change its fate [126].

### Phagocytosis of non-apoptotic cells

Efferocytosis is defined as the engulfment and digestion of dying cells by phagocytes [130]. It has been shown that, while phagocytosis is PS-dependent in both apoptotic and necrotic cells, the later are phagocytosed less quickly and efficiently [100]. Recently, our group has shown that AnnexinV+ necroptotic U937 cells are phagocytosed by BMDMs and peritoneal macrophages more efficiently than live cells [103]. In support, phagocytosis of necroptotic Jurkat cells was observed while their plasma membrane was still intact [116]. Budai et al. recently reported that apoptotic and necrotic cells are equally engulfed. The phagocytosis in both cases is still PS-dependent, as it was reduced by masking PS, or by deficiency in the PS-receptors: T-cell immunoglobulin mucin protein-4 (TIM4), Mer receptor tyrosine kinase (MerTK), integrin *β* 3, and tissue transglutaminase (TG2) [131]. The type of engulfed and engulfing cells, as well as the molecular mechanisms or duration of PS exposure, might all contribute to these observations.

As mentioned above, CDC50A-deficient cells constitutively expose PS. These cells, although live, are engulfed by wild-type, but not MerTK-deficient, macrophages, indicating that PS is sufficient to induce phagocytosis. Interestingly, 3% of the engulfed live cells are released intact, a phenomenon that is not seen in apoptotic cells with active capsases [108]. In contrast, the same group has reported that live cells continually exposing PS due to constitutively active TMEM16F are not engulfed by macrophages, suggesting that the mechanism of PS exposure might influence the consequent phagocytosis [132].

A metabolically stressed cell uses classical autophagy, an evolutionarily conserved pathway, as a source of nutrients. MAPPLC3A (LC3), which has an essential role in the classical autophagy pathway, was found to have a key role in a similar, but distinct, pathway – LC3-associated phagocytosis, or LAP. Uptake of either apoptotic, necrotic, or necroptotic cells was shown to promote LAP, characterized by the translocation of LC3 to the phagosome. This consequently facilitates phagosome maturation and the degradation of the engulfed dead cells. LAP was mediated by PS recognition by the receptor TIM4, as TIM4-deficient macrophages failed to undergo LAP [133]. LAP-deficient mice exhibit normal engulfment, but defective degradation, of apoptotic cells. Upon repeated injection of apoptotic cells, these mice developed a systemic lupus erythematosus (SLE)-like disease, with increased levels of pro-inflammatory cytokines, such as IL-6, IL-1*β*, IL-12, autoantibodies, and a decreased level of the anti-inflammatory cytokine, IL-10. These data are consistent with the notion that defects in the clearance of dying cells underlie the pathogenesis of SLE [134]. In addition, LAP-deficiency in tumor-associated macrophages (TAM) triggers pro-inflammatory and stimulator of interferon gene (STING)-mediated type I interferon gene expression in response to phagocytosis of apoptotic cells, in contrast to an M2 phenotype seen in the wild-type TAMs. In support, defects in LAP in the myeloid compartment induce a type I interferon response and suppression of tumor growth [135]. This suggests that phagocytosis can be regulated downstream of PS-mediated engulfment, leading to different effects. Taken together, these reports have implications for how we define apoptosis as an immunologically silent process in contrast to other non-apoptotic forms of cell death, and strongly suggest our current model for PS exposure during cell death is overly simplistic. Overall, these studies highlight how much is yet to be uncovered regarding the contribution of PS to downstream signaling in cell death.

### The role of PS-positive non-apoptotic cells and EVs

Given that non-apoptotic cells are known to expose PS and be phagocytosed, albeit via a not-yet-fully-defined mechanism, the immunological consequences for non-apoptotic cell death should be re-examined. As discussed, death of PS-exposing necroptotic cells can be leashed by the ESCRTIII-mediated shedding of PS-exposing bubbles to maintain plasma membrane integrity [102, 103, 116, 120, 121, 126]. In support, during pyroptosis the ESCRT machinery, in association with gasdermin-D, is seen to be recruited to damaged membranes to induce the budding of AnnexinV+ vesicles and negatively regulate death [136]. Hence, the phase in which cells expose PS could be viewed as a ‘window of opportunity’ for the cell to manipulate inflammatory cell death pathways, and potentially control the release of pro-inflammatory DAMPs and cytokines, such as IL-1*β* in pyroptosis [137] and IL-33 in necroptosis [138]. Additional support for the immuno-regulatory role of PS exposure is that mice lacking the phospholipid scramblase, XKR8, exhibited reduced clearance of apoptotic lymphocytes and neutrophils, and an SLE-like autoimmune disease [139]. However, XKR8 activity is caspase-dependent and, thus, most likely inactive during necroptosis [140]. Deficiency of TMEM16F has not been reported to induce the same autoimmune disease, but does result in a mild bleeding disorder associated with the role of PS in activated platelets. This fits with a splice mutation in *TMEM16F* found in patients with a similar bleeding disorder, named Scott’s syndrome [141, 142]. Filling in the gaps in our understanding of the biology of PS exposure by non-apoptotic cells might reveal how this system is modulated under different conditions to fine-tune the downstream immune response.

The necroptotic factors, RIPK1, RIPK3, and MLKL, induce expression of inflammatory cytokines and chemokines [143–148]. PS-exposing necroptotic cells lacking ESCRTIII components have reduced expression and release of these cytokines and chemokines. In addition, while necroptotic cells potently induce cross-priming of CD8^+^ T cells via RIPK1 and NF-kB [149], this is reduced in ESCRTIII-deficient cells [102]. In support, Kearney et al. have reported that necroptotic death attenuates production of pro-inflammatory cytokines and chemokines by lipopolysaccharide (LPS) or TNF [150]. These results suggest that the ESCRT-driven delay in cell death execution, mediated by repair of PS-exposing membrane, enables a sustained time for inflammatory signaling. This highlights that the time interval associated with PS exposure, rather than the cell lysis itself, might be the inflammation-promoting arm of necroptosis.

Reports regarding the sequential events in the phagocytosis of dying cells are somewhat confusing. Phagocytosis of apoptotic cells by LPS-activated monocytes has been reported to increase IL-10 secretion, while reducing secretion of TNF-α, IL-1*β*, and IL-12 [151]. In addition to IL-4 and IL-13, recognition of apoptotic, but not necrotic, neutrophils by the PS-receptors MerTK and Axl is essential for induction of anti-inflammatory and repair programs in BMDMs [152]. We have also shown that phagocytosis of both PS-exposing apoptotic and necroptotic cells results in IL-6 secretion, while only phagocytosis of necroptotic cells leads to significantly elevated TNF-α and CCL2 secretion from macrophages [103]. Necroptotic cancer cells induce dendritic cell maturation in vitro, cross-priming of T cells in vivo, and antigen-specific IFN-*γ* production ex vivo. Vaccination with necroptotic cancer cells facilitates efficient anti-tumor immunity [153], and administration of mRNA coding for MLKL induces anti-tumor immunity [154, 155]. Martinez et al. have reported that phagocytosis of either apoptotic, necroptotic, or necrotic cells is followed by the secretion of IL-10 (higher in apoptosis) and transforming growth factor (TGF)-*β* (slightly higher in necroptosis). LAP-deficient macrophages secrete elevated levels of IL-1*β* and IL-6, but show decreased IL-10 and TGF-*β*, in response to these dying cells [133]. This is consistent with the anti-tumor or auto-immunity seen when LAP is impaired, further implicating LAP in the regulation of the immune response [133–135].

As previously proposed in our model of the ‘three waves of immunomodulatory effects during necroptosis’, the PS-exposing bodies released during early necroptosis may serve as signaling vehicles that stimulate the microenvironment [120, 126]. For example, EVs that are released from LPS-activated, caspase-8-deficient BMDMs in a MLKL-dependent manner, contain IL-1*β* [121]. In addition, the fact that phagocytosis of necroptotic, but not apoptotic, cells induces inflammation might be explained by the presence of necroptotic bodies, rather than a distinct effect of these PS-exposing engulfed cells.

### Concluding remarks

Exposure of PS by non-apoptotic cells has long been disregarded, leading to the role of PS exposure during apoptosis being overstated with respect to how inflammation is mitigated during apoptosis. Here, we have briefly outlined apoptotic and necroptotic RCD, and their respective roles in promoting inflammation. We have outlined the evidence for PS exposure in non-apoptotic cells and EVs, discussed a potential mechanism, and looked at the effect of PS-exposure on the reversibility of cell death, the phagocytosis of dead cells, and subsequent inflammation.

Recent reports challenging the idea that PS exposure is exclusive to apoptosis highlight that communication between RCD and the immune system is far from being fully understood. Even more fundamental, however, is the need to improve the classification of RCD pathways in published literature, as well as develop more definitive methods for their characterization. As non-apoptotic cells can also present “eat me” signals and be engulfed, phagocytosis should be considered as a kind of ‘bridge’ between a dying cell and the immune system. How dying cells affect signaling in phagocytes will be fascinating to examine in light of this new understanding. In this regard, studying the contents, uptake, and dissemination of PS-exposing vesicles may shed light on the immunological effects of non-apoptotic RCD. In addition, a better understanding of PS exposure and recognition of non-apoptotic cells by phagocytes might provide new therapeutic tools in the PS field. The evident involvement of the ESCRTIII machinery could be manipulated as a powerful tool to regulate cell death and inflammation. In examining PS biology, this review challenges the dichotomy typically thought to exist between apoptosis and other forms of RCD, and highlights the importance of understanding the inflammatory consequences of PS exposure in the context of all cell death modalities.

## Data Availability

Not applicable.

## Abbreviations

ABC: ATP-binding cassette
ACD: Accidental cell death
AD: Alzheimer’s disease
AD: Anno Domini
AIM2: Absence in melanoma 2
ALS: Amyotrophic lateral sclerosis
ANOH-1: Anoctamin homolog-1
Bcl-2: B-cell lymphoma-2
BMDCs: Bone marrow-derived dendritic cells
BMDMs: Bone marrow-derived macrophages
Ca: Calcium
c-FLIP: Cellular FLICE (FADD-like IL-1β-converting enzyme)-inhibitory protein
cIAPs: Cellular inhibitor of apoptosis
CMV: Cytomegalovirus
DAI: DNA-dependent activator of IFN-regulatory factors
DAMPs: Danger associated molecular patterns
DD: Death domain
DNA: Deoxyribonucleic acid
dsRNA: Double stranded ribonucleic acid
EBV: Epstein-Barr virus
EPEC: Enteropathogenic *Escherichia coli*
ER: Endoplasmic reticulum
ESCRT: Endosomal sorting complexes required for transport
EVs: Extracellular vesicles
FADD: Fas-associated protein with death domain
FASL: Fas ligand
FGFR: Fibroblast growth factor receptor
GPX4: Glutathione peroxidase 4
HSV: Herpes simplex virus
IFN: Interferon
IFNR: IFN receptors
IL: Interleukin
IRF: Interferon regulatory factor
JAK: Janus kinase
LAP: LC3-associated phagocytosis
LC3: MAPPLC3A
LPS: Lipopolysaccharide
LUBAC: Linear ubiquitin chain assembly complex
MAVS: Mitochondrial antiviral-signaling protein
MerTK: Mer receptor tyrosine kinase
MLKL: Mixed lineage kinase domain-like
MOMP: Mitochondrial outer membrane permeabilization
MPT: Mitochondrial permeability transition
MS: Multiple sclerosis
NF-kB: Nuclear factor kappa-light-chain enhancer of activated B cells
NSA: Necrosulfonamide
P4-ATPase: Type IV P-type ATPase
PAMPs: Pathogen associated molecular patterns
PBL: Peripheral blood lymphocytes
PE: Phosphatidylethanolamine
pMLKL: phosphorylated MLKL
PS: Phosphatidylserine
RCD: Regulated cell death
RIG-I: Retinoic acid-inducible gene I
RIPK1: Receptor-interacting serine/threonine-protein kinase 1
RIPK3: Receptor-interacting serine/threonine-protein kinase 3
SLE: Systemic lupus erythematosus
SMAC: Second mitochondria-derived activator of caspases
STAT: Signal transducer and activator of transcription
STING: Stimulator of interferon genes
TAM: Tumor associated macrophages
TG2: Tissue transglutaminase
TGF: Transforming growth factor
TIM4: T cell immunoglobulin mucin protein-4
TLRs: Toll-like receptors
TMEM16F: Transmembrane protein 16F
TNF: Tumor necrosis factor
TNFR: TNF receptor
TRADD: TNFR type 1-associated via death domain
TRAF2: TNF receptor associated factor 2
TRAIL: TNF-related apoptosis-inducing ligand
TRIF: TIR-domain-containing adapter-inducing interferon- *β*
XKR8: XK-related protein 8
